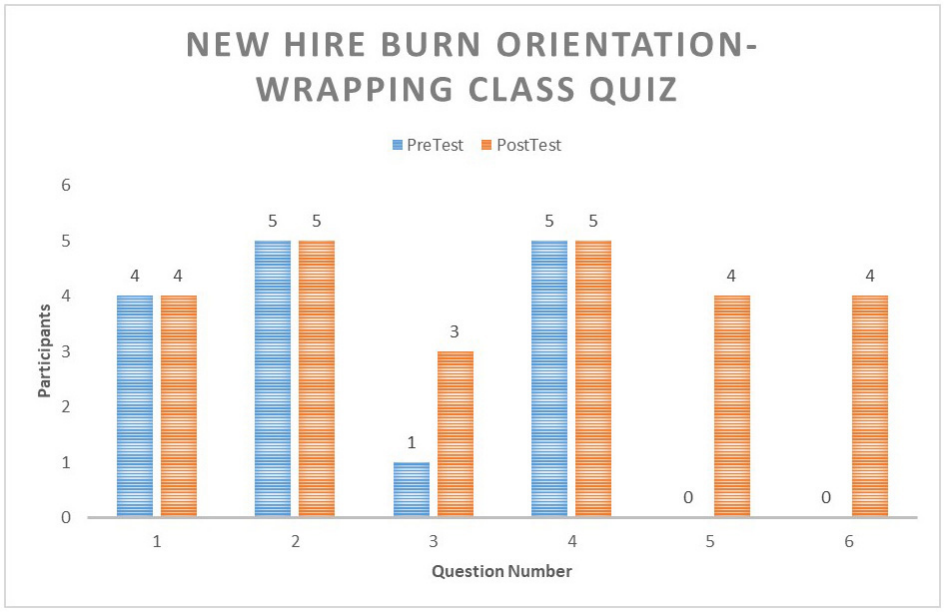# 95 Clinical Advisor-Led Dressing Class to Enhance New Graduate Nurse Pediatric Burn Knowledge and Perceived Relevance

**DOI:** 10.1093/jbcr/iraf019.095

**Published:** 2025-04-01

**Authors:** Lisa Shostrand, Erin Kramer, Brett Hartman

**Affiliations:** Riley Children’s Health; Riley Hospital for Children; Eskenazi Health

## Abstract

**Introduction:**

New graduate nurses receive limited pediatric burn background information and/or direct practice with ointments, dressings, and other burn products prior to unit shifts as part of standard orientation. To enhance opportunities for clinical growth, a burn dressing class was developed and taught by a core group of pediatric burn nurse advisors to new graduate nurses. This experiential learning session entailed a pre- and post-questionnaire in addition to a class evaluation to assess knowledge and relevance.

**Methods:**

A six-hour class created and led by two burn clinical advisors was taught to five new burn nurses. A six-question multiple choice test was administered to the participating nurses immediately pre- and post- instruction. A dressing skills validation was also completed after an instructor-led hand and head wrapping demonstration. Teach back was the method utilized to ensure learning had occurred. At the conclusion of the class, an evaluation assessing relevance and understanding was distributed.

**Results:**

Results demonstrated the nurses increased their knowledge from the pre- to post-assessment by 40%. The nurses completed the head and hand wrapping skills validations with 100% accuracy. Four of the five participants completed the post-class evaluation. All felt the class was relevant to current practice, while 50% stated they could now be successful and had a solid understanding of the concepts taught. All participants shared they were either completely riveted or their attention rarely wandered during the presentation.

**Conclusions:**

For this new pediatric burn nurse group, results illustrated learning occurred and practice relevance was appreciated immediately after the class concluded. This score improvement demonstrated a knowledge and application gap in burn care in the new graduate burn nurse orientation which was addressed by this course. Assessment from a competency lens or organizational perspective would be the next step for higher level evaluation.

**Applicability of Research to Practice:**

Pediatric burn nursing is a specialty field with which many new graduate nurses have no background knowledge or experience. By creating a pediatric-specific burn course, new nurses can have the chance to learn, apply and demonstrate burn wrapping skills prior to completing orientation. This instruction may be impactful on future research of orientation best practices as well as learning needs of new pediatric burn nurses.

**Funding for the Study:**

N/A